# Effect of pretreatment with Devil's Claw on locomotor activity, infarct volume, and neuronal density in focal cerebral ischemia in rats

**DOI:** 10.22038/AJP.2024.24294

**Published:** 2024

**Authors:** Shima Shirzad, Mona Riyahi Rad, Mohammad Rezaei, Mitra Tayaranian Marvian, Arman Abroumand Gholami, Fatemeh Forouzanfar, Mansoureh Sabzalizadeh, Hamed Ghazavi, Farzaneh Vafaee

**Affiliations:** 1 *Neuroscience Research Center, Mashhad University of Medical Sciences, Mashhad, Iran *; 2 *Department of Neuroscience, Faculty of Medicine, Mashhad University of Medical Sciences, Mashhad, Iran*; 3 *Department of Cellular Biology and Anatomical Sciences, School of Medicine Mashhad University of Medical Sciences, Mashhad, Iran*; 4 *Neuroscience Research Center, Institute of Neuropharmacology, Kerman University of Medical Sciences, Kerman, Iran*

**Keywords:** Cerebral ischemia, Devil's Claw, Locomotor activity, Neuronal density

## Abstract

**Objective:**

Stroke is a highly prevalent and devastating condition affecting millions worldwide. The Devil's Claw (DCW) plant is a native African plant whose anti-inflammatory, antioxidant, and neuroprotective properties have been investigated. We postulated that DCW could protect the brain injury caused by cerebral ischemia.

**Materials and Methods:**

The rats were randomly divided into four groups. The sham and control (Ctrl) groups received pretreatment with a distilled water vehicle. Doses of 200 and 400 mg/kg were selected for pretreatment with DCW. The filament or intravascular occlusion method was used for middle cerebral artery occlusion (MCAO). The Triphenyl tetrazolium chloride (TTC) staining method was used to investigate the infarct zone and penumbra volume. The neuroprotective effect of DCW was measured by hematoxylin staining. Movement performance was evaluated from neurological deficit score, rotarod performance, and open field tests.

**Results:**

TTC staining showed that the DCW/400 group could maintain the penumbra's structure and reduce the infarct volume compared to the Ctrl group (p<0.001). Histological studies confirmed the neuroprotective properties of DCW at doses of 200 and 400 mg/kg compared to the Ctrl group (p<0.01 and p<0.0001, respectively). The results of behavioral tests showed an improvement in behavioral performance in pretreatment 400 mg/kg doses compare to Ctrl group (p<0.0001).

**Conclusion:**

The study showed that pretreatment with DCW with its neuron protection potential reduces the infarct area and restores motor function after MCAO.

## Introduction

Stroke is a significant cause of death and disability in modern society, affecting many individuals yearly (Pulatov et al., 2022 ▶; Wang et al., 2020 ▶). It results in significant costs to society and leads to lifelong disability. Unfortunately, no effective treatment has been found yet. Pathological conditions such as cerebral ischemia affect all neurovascular elements, including neurons, astrocytes, microglia, endothelial cells, and vascular wall cells (Abroumand Gholami et al., 2023 ▶; Ghazavi et al., 2020 ▶; McCrary et al., 2020 ▶; Shirzad et al., 2024 ▶). Inflammation is one of the main pathophysiological mechanisms of stroke. Activated microglia produce large amounts of proinflammatory cytokines, toxic metabolites, and enzymes. Astrocytes also play an important role in stroke inflammation (Stadler et al., 2022 ▶). 

Moreover, the reduced blood supply of adenosine triphosphate (ATP) to the brain impairs mitochondrial function, producing oxygen free radicals and peroxidation of cell membrane lipids (Azam et al., 2019 ▶). This condition triggers oxidative stress in the lesion and inflammation, resulting in apoptosis and inflammatory responses (Ciancarelli et al., 2022 ▶). These are often associated with a blood-brain barrier (BBB) disorder, followed by cerebral edema (Ciancarelli et al., 2022 ▶; Jin and Leng, 2022 ▶; McCrary et al., 2020 ▶; Stadler et al., 2022 ▶). Therefore, these pathological processes could be a therapeutic target in stroke.

Devil’s Claw (DCW), or Harpagophytum* procumbens*, belongs to the sesame family and is native to South Africa. Natives have traditionally used it as an anti-inflammatory folk remedy for fever, indigestion, malaria, allergies, skin cancer, rheumatism, and arthritis (Mariano et al., 2022 ▶; Menghini et al., 2019 ▶; Peruru et al., 2020 ▶). DCW has been found to possess anti-inflammatory and anti-apoptotic properties through the inhibition of interleukin-6 (IL-6) and tumor necrosis factor alpha (TNF-α), resulting in the downregulation of nuclear factor kappa B (NF-κB) expression and modulation of the apoptotic pathway dependent on mitogen-activated protein kinases (MAPKs) and phosphoinositide-3-kinase–protein kinase B/Akt (PI3K-PKB/Akt) (Fiebich et al., 2012 ▶; Koycheva et al., 2021 ▶; Menghini et al., 2019 ▶). Furthermore, the primary chemical components found in DCW, which include scersoside, chromonoside, harpagoside, saponins, cystrines, and alkaloids, have been identified as the primary factors responsible for reducing levels of free inflammation and pain associated with central nervous system diseases by inhibiting the activity of cyclooxygenase-2 (COX-2) and lipoxygenase (LOX), which are enzymes involved in the inflammatory cascade (Huang et al., 2006 ▶). Moreover, treatment with only the DCW plant led to increased activity of antioxidant factors in the brains of rats, resulting in enhanced neuronal survival (Georgiev et al., 2010 ▶). 

Taken together, the findings from the studies mentioned above suggest that DCW may be promising as a potential therapeutic agent for mitigating the pathophysiological mechanisms of stroke by targeting specific molecular pathways and the properties of its chemical constituents. Nonetheless, there remain to be more studies investigating the efficacy of DCW in treating neurological disorders, particularly stroke. Therefore, this study aimed to investigate the effects of the Devil's Claw plant on motor activity, lesion volume, and neuronal density in the penumbra region.

## Materials and Methods

**Animals and housing**

All procedures related to animals were approved by the Animal Ethics Committee of Mashhad University of Medical Sciences (Mashhad, Iran) and were carried out following the National Institute of Health guidelines for the care and use of laboratory animals and the guidelines of the Iranian Institutional Animal Ethics Committee (ethics code: IR.MUMS.MEDICAL.REC.1400.100). The adult male Wistar rats (250-280 g) used in this study were kept in the animal room of Mashhad University of Medical Sciences (MUMS) under standard conditions with a 12-hour light/dark cycle and an almost constant temperature of 22±2°C with free access to food and water. 

**Drug administration**

The dry powder of DCW extract was purchased from Razak Co. (Iran). DCW powder was dissolved in distilled water. Groups pretreated with DCW received doses of 200 and 400 mg/kg of the drug daily for three weeks before surgery. Other study groups received distilled water.

**Experimental design**

The animals were pretreated by daily gavage for three weeks before the operation. Thirty-two rats were randomly assigned to four groups (n=8) as described following: (1) The Sham group underwent a sham operation and received distilled water as the vehicle orally (Sham), (2) the control operated group that underwent middle cerebral artery occlusion (MCAO) and received distilled water as the vehicle orally (Ctrl), (3) the MCAO group that received DCW pretreatment at 200 mg/kg doses (DCW/200), and (4) the MCAO rats that received DCW pretreatment at 400 mg/kg doses (DCW/400).

**Induction of MCAO**

Animals were anesthetized by intraperitoneal injection of chloral hydrate (450 mg/kg) (Kolpakova et al., 2017 ▶). Before surgery, 2 mg/kg meloxicam was injected subcutaneously as an analgesic. Induction of MCAO was performed by a filament or intravascular occlusion method (Ashioti et al., 2009 ▶). Briefly, a longitudinal incision was made in the midline of the neck to expose the right common carotid artery. A 3-0 sterile monofilament nylon suture with a silicone coat was inserted into the intracranial distal of the internal carotid artery. It was advanced approximately 17.5 mm from the carotid incision site to obstruct the blood flow to the middle cerebral artery (MCA). Following 30 minutes of MCAO, the filament was retracted completely to allow reperfusion of the MCA. Then the incision site was sutured and disinfected. The animals were kept under warm lamps until they woke up.

**Behavioral studies**

**Neurological deficit score (NDS)**

Examination of NDS in all groups is done one day after surgery. This evaluation measured six parameters, including spontaneous activity, symmetry in the movement of four limbs, forepaw outstretching, climbing, body proprioception, and response to vibrissae touch. The score given to each rat at the end of the evaluation is the sum of all six individual test scores. The neurological score ranged from 3 to 18 (Garcia et al., 1995 ▶).

**Rotarod performance test (RPT)**

We used the rotarod performance test to measure the movement coordination of the animals by recording the time they stayed on the cylindrical rotarod. The animals are trained on the rotarod device for three days before the surgery, with three sessions daily (from 4 to 40 rpm in 5 minutes). The animals are tested one day before and after the surgery, and the time they stay on the rotarod is recorded. We test the animals twice with a 15-minute break between the trials. The average of two experiments was calculated for each rat. RPT data were presented as the mean percentage of time on the rotarod compared to the sham group (before surgery).

**Open field test**

We used the open field test (OFT) to assess the motor impairments after the injury. In this test, we put each rat in the middle of an open field with a box (60 cm L × 45 cm W × 45 cm H) in a dim and quiet room. Movement activity was measured by counting the rats' total crossing and distance traveled. The total crossing was the number of grid lines the rodent crossed with all four paws, and the total distance traveled was the amount of distance the rat moved while standing on its hind paws. Total distance traveled and crossing for 10 minutes one day after surgery were recorded. A videotape was used to track the animals, and a blind assessor was used to analyze them.

**Infarct volume quantification**

Histological assessments were carried out on all experimental groups one day following the induction of MCAO (n=3). Briefly, all animals were intraperitoneally anesthetized with ketamine (100 mg/kg) and xylazine (10 mg/kg). Then, a normal saline solution was injected through the left ventricle while the abdominal aorta was clamped to provide cerebral perfusion. Subsequently, segmented brain tissues (2 mm) were subjected to a 2% triphenyltetrazolium chloride (TTC) staining process, which lasted for 25 min at 37 degrees C. This process facilitated the differentiation of metabolically active and infarcted tissue, with living brain tissue cells appearing in red and damaged or dead cells appearing in white. 

In order to quantify the extent of the infarct, we utilized a modified formula for calculating infarct volume. This formula obtained the infarct's total volume by multiplying the brain slices' average thickness by the infarct area's sum across all slices. The modified infarct volume was then calculated using the formula: Infarct area = Contralateral hemisphere area– Healthy area of ipsilateral hemisphere. Infarct volume = Infarct area × Thickness of slice

**Histological determination of neuronal density**

For histological examinations one day after surgery, we sacrificed the remaining animals in all groups with deep anesthesia and then perfused them with 10% formalin (n=5). Then, after collecting the brains and forming paraffin blocks (tissue location: 1.9-3.1 mm posterior to the bregma), we randomly prepared coronal tissue sections using a Rotary microtome - MR 3000 (Histo-Line, Italy) with a thickness of 5 μm. Next, we performed histological differentiation of the sections to identify the neurons based on hematoxylin staining. We studied different areas under a light microscope with a camera (BX51, Olympus, Japan) and took images under an objective lens (X10 and X40; Olympus, Japan). For the stereological method, we randomly took five photos from each slide from 5 different points (4 corners and one center). Then we graded the image with 2 x 2 squares. Next, we randomly selected six squares from the gridded squares and counted the number of viable neurons in these six squares in all images. Next, we calculated neuron counts using the stereological software package (Mosaic Software, USA). Finally, we calculated the total number of counted cells from the formula: ND=ΣQ/Σframe × A frame × H

The ND, ΣQ, Σframe, A- frame, and H represent total neuronal density, the sum of neurons counted in a sample, the total number of times sampled in one sample, the counting frame area, and the distance between two consecutive slices, respectively. Finally, the number of live neurons was measured based on the percentage of live neurons compared to the sham group.

**Statistical analysis**

The group size was determined based on a power calculation previously described in García-Bonilla et al. (2011). Statistical analysis was conducted using the statistical software for GraphPad Prism version 9 (San Diego, CA, USA). Data were analyzed using one-way ANOVA with the Tukey post hoc test for multiple group comparisons. In addition, the behavioral scores for NDS and total-cross test were analyzed using the Kruskal-Wallis test with Dunn's post hoc test for multiple group comparisons. Finally, RPT was analyzed using two-way ANOVA multiple comparisons between groups. Data are presented as mean±standard deviation (SD). The significance was at p<0.05.

## Results

**Effect of DCW pretreatment on neurobehavioral function after MCAO**

**Figure 1:**
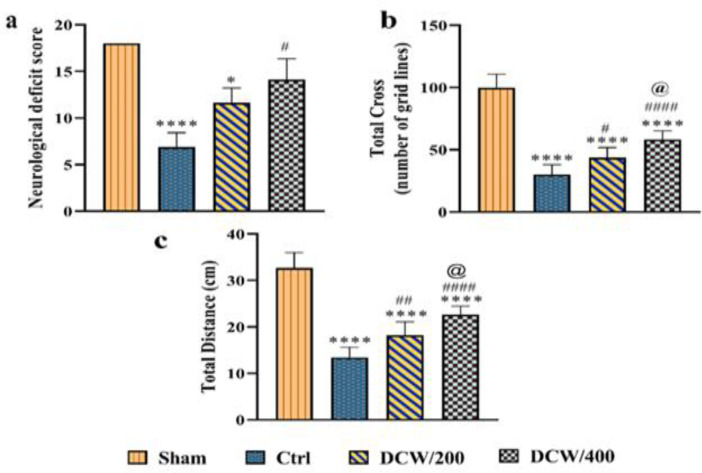
The effect of DCW pretreatment on animals' neurological deficit score (NDS) and (b and c) exploratory behavior one day after MCAO induction. The presented values represent the mean and SD (N=8). *, # and @ indicate differences compared to Sham, Ctrl, and DCW/200 groups, respectively (*p<0.05, ****p<0.0001, #p<0.05, ##p<0.01, ####p<0.0001, and @p<0.05). The data from NDS and total-cross were analyzed using the Kruskal-Wallis test, and the total distance was analyzed using the one-way ANOVA. Ctrl; the control operated group that underwent middle cerebral artery occlusion, DCW/200; the MCAO group that received DCW pretreatment at 200 mg/kg doses, DCW/400; the MCAO group that received DCW pretreatment at 400 mg/kg doses

Animals were evaluated regarding neurological deficits at 24 hr, and the corresponding functional score was obtained for each group (Figure 1). No neurological deficits were detected in the sham group. On the NDS scale, a significant difference was observed between the Ctrl and sham groups (p<0.0001). The DCW/400 group showed good functional improvement compared to the Ctrl group (p<0.05), without any significant difference in functional scores with the sham group. However, no significant difference existed between the DCW/200 group with the Ctrl and DCW/400 groups (Figure 1 a and b).

The sham group showed a normal level of activity in OFT. Pretreatment with DCW/400 could significantly increase the total cross and distance traveled compared to the Ctrl group (p<0.0001 and p<0.0001, respectively). However, the total cross and distance traveled decreased significantly in all groups compared to the sham group (p<0.0001 for all groups). Pretreatment with DCW was able to increase the level of these activities compared to the Ctrl group after the MCAO (p<0.0001 and p<0.0001 for DCW/400 group and p<0.01 and p<0.05 for DCW/200 group, respectively). 

**Figure 2:**
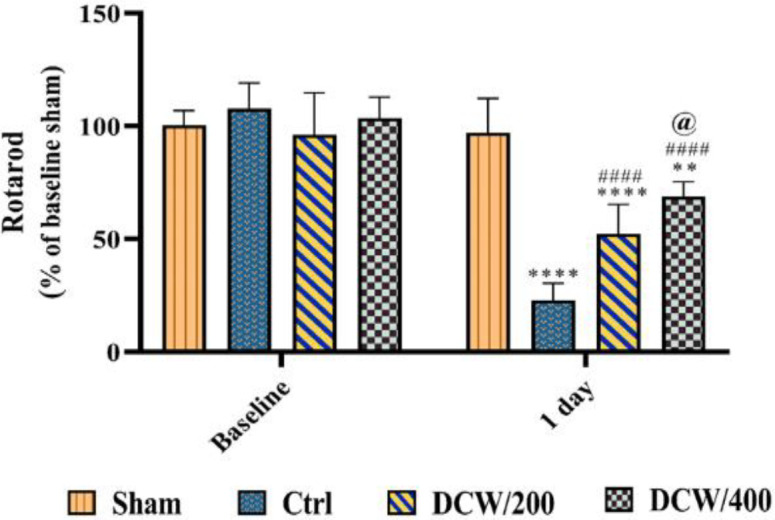
The graph shows the behavioral performance of the study groups one day before and after the MCAO through the rotarod performance test. *, # and @ indicate differences compared to Sham, Ctrl, and DCW/200 groups, respectively (**p<0.01, ****p<0.0001, ####p<0.0001, and @p<0.05). Data were analyzed using two-way ANOVA. Ctrl; the control operated group that underwent middle cerebral artery occlusion, DCW/200; the MCAO group that received DCW pretreatment at 200 mg/kg doses, DCW/400; the MCAO group that received DCW pretreatment at 400 mg/kg doses

Also, the DCW/400 group has shown better recovery than the DCW/200 group by increasing this exploratory movement (p<0.05) (Figure 1b and c).The results of the RPT showed that there was no significant difference among the groups before the induction of the model (Figure 2). After MCAO induction, despite the significant difference among all groups with the sham group (p<0.0001, p<0.0001, and p<0.01 for Ctrl, DCW/200, and DCW/400, respectively), both groups pretreated with DCW were able to increase their behavioral performance compared to the Ctrl group (p<0.0001 and p<0.0001 for DCW/200 and DCW/400; respectively). Also, improved behavioral performance in the DCW/400 group was significantly higher than the DCW/200 group (p<0.05).

**Figure 3:**
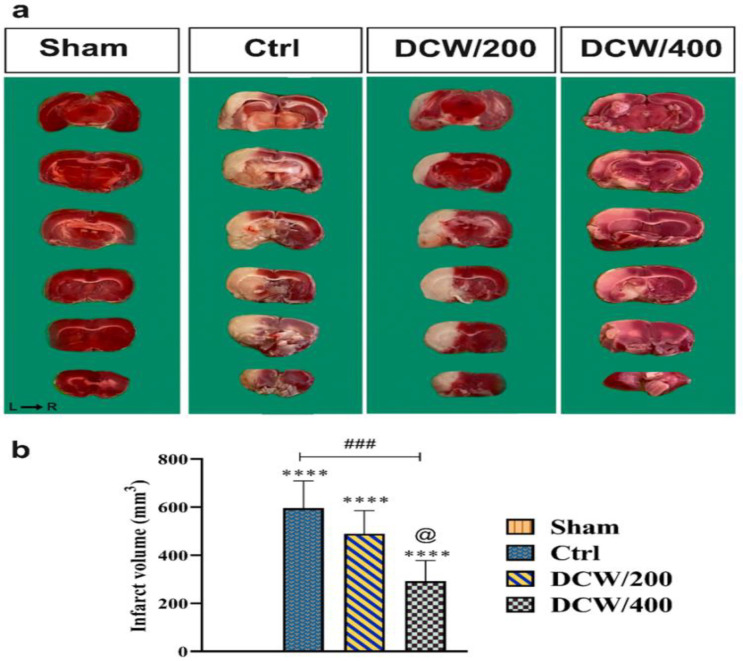
(a) Brain segments stained with triphenyltetrazolium chloride (TTC). (b) The diagram shows the difference between TTC-stained groups. *, # and @ indicate differences compared to Sham, Ctrl, and DCW/200 groups, respectively (****p<0.0001, ##p<0.01, ####p<0.0001, and @p<0.05). The data were analyzed using one-way ANOVA. Ctrl; the control operated group that underwent middle cerebral artery occlusion, DCW/200; the MCAO group that received DCW pretreatment at 200 mg/kg doses, DCW/400; the MCAO group that received DCW pretreatment at 400 mg/kg doses

**DCW pretreatment reduces the macro- and micro-histological changes induced by MCAO**

TTC staining is a method that is generally used to assess the volume of the infarct region. Figure 3 shows the macro-anatomy images of sections stained with TTC and the infarct volume diagram. The results of the macro-anatomical images show that pretreatment with DCW reduced the infarct volume (Figure 3a). In addition, the diagram results showed that the DCW/400 group significantly reduced the infarct volume compared to the Ctrl and DCW/200 groups (p<0.001 and p<0.0001, respectively; Figure 3b).

**Figure 4:**
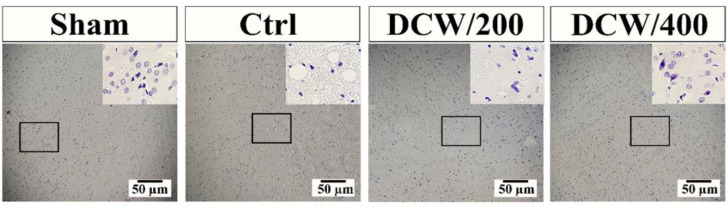
The images of sections stained with hematoxylin showed that pretreatment with DCW could preserve the tissue's microanatomical structure with its neuronal protection properties. The images with the upper area are at 10x magnification, and the lower area images are at 40x magnification for each group. Ctrl; the control operated group that underwent middle cerebral artery occlusion, DCW/200; the MCAO group that received DCW pretreatment at 200 mg/kg doses, DCW/400; the MCAO group that received DCW pretreatment at 400 mg/kg doses

**Figure 5:**
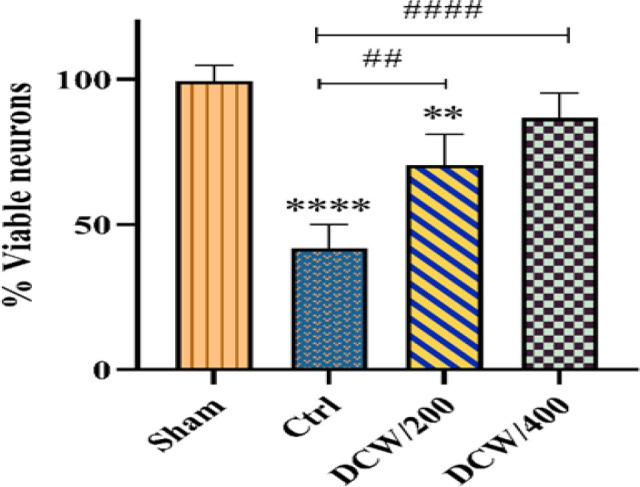
The diagram indicates the difference between the groups stained with hematoxylin. The presented values represent the mean and SD (n=5). * and # indicate differences compared to Sham and Ctrl groups, respectively (**p<0.01, ****p<0.0001, ##p<0.01, and ####p<0.0001). The data were analyzed using one-way ANOVA. Ctrl; the control operated group that underwent middle cerebral artery occlusion, DCW/200; the MCAO group that received DCW pretreatment at 200 mg/kg doses, DCW/400; the MCAO group that received DCW pretreatment at 400 mg/kg doses

Figure 4 and 5 represents the density of neurons in the penumbra region and the diagram of the % viable neurons in the studied groups. As shown in Figure 4, pretreatment with DCW has a neuron protection effect at the penumbra site. The diagram results indicate that the groups pretreated with DCW have more neuronal protection than the Ctrl group after MCAO (p<0.0001). Although no significant difference was seen between the two pretreated groups, the DCW/400 group was able to bring the changes to the level of the sham group (Figure 5).

## Discussion

The findings of our study indicate that the extent of recovery from MCAO-induced deficits depends on the dosage of DCW pretreatment. Specifically, at three weeks post-gavage of 200 mg/kg or more DCW, there was a significant improvement in most behavioral performance one day after MCAO. In addition, macro- and micro-anatomy changes significantly improved in the 400 mg/kg dose DCW. This condition is the first report to our knowledge that demonstrates the ability of DCW to preserve neuron density and reduce infarct volume after stroke, mainly when administered at a dose of 400 mg/kg DCW. In instances where blood circulation to a particular brain region is impeded, transmissions of nerve impulses from brain neurons experience interference, leading to eventual nerve cell degradation in the affected area, with strokes particularly implicated (Pulatov, 2022 ▶).

Concerning MCAO, the cerebral infarct area enlarges alongside corresponding increased functional impairments in the patient (Wang et al., 2020 ▶). The penumbra zone undergoes a decline in the response capacity consequent to neuronal destruction. This reaction also interferes with signaling pathways conducive to regenerating new nerve cells (McCrary et al., 2020 ▶). Studies have revealed that inflammation and oxidative stress can impede the neuronal plasticity of the penumbra in stroke, resulting in infarct expansion (Ciancarelli et al., 2022 ▶; Sabahi et al., 2022 ▶). The DCW plant is characterized by its anti-inflammatory, analgesic, and antioxidant properties, corroborated by various investigations (Grąbkowska et al., 2016 ▶; Menghini et al., 2019 ▶). It has been further demonstrated that administering the DCW inhibits gliosis and helps counter the destruction of neuron nuclei in the model of arsenic neurotoxicity (Peruru et al., 2020 ▶). The anti-inflammatory potential of the DCW is credited to iridoid glycosides, while its antioxidant effects are attributed to flavonoid and phenolic compounds (Grąbkowska et al., 2016 ▶; Mariano et al., 2022 ▶). 

The findings of our study suggest that administering DCW prior to a stroke event can reduce the volume of infarct, resulting in improved performance in behavioral tests. Harpagoside, a key iridoid found in DCW, has been found to increase glutathione peroxidase and decrease lipid peroxidation through inhibition of the NF-kB pathway in the brains of rats (Huang et al., 2006 ▶). Additionally, harpagoside has been shown to increase norepinephrine and serotonin levels by modulating oxidative stress in an Alzheimer's model (Menghini et al., 2019 ▶). In our study, we induced stroke and evaluated the resulting neurobehavioral changes in rats using various behavioral parameters. The MCAO procedure progressively suppresses animal behavior, as evidenced by functional status, neurological impairments, locomotor performance, and exploratory locomotion measured by distance traveled and crossing lines. Curiously, pretreating rats with DCW at 400 mg/kg dose almost normalized these experimental parameters. These beneficial effects of DCW pretreatment suggest that it may possess anti-infarct properties. As reported in previous studies, the observed behavioral recovery and reduction in infarct volume may be explained by the preservation of dopaminergic receptors and maintenance of neurotransmitter levels by DCW (Mahomed and Ojewole, 2006 ▶; Sun et al., 2012 ▶). However, neurotransmitter levels were not investigated in our study. Further research is necessary to elucidate the underlying mechanism of DCW in limiting the infarct area.

The induction of MCAO increases the production of free radicals, which results in oxidative stress in living organisms (Jin and Leng, 2022 ▶). Consequently, this process can cause peroxidation of membrane lipids, leading to the loss of membrane integrity and function (Lu et al., 2023 ▶). Our study demonstrates that DCW possesses neuron-protective properties, as evidenced by preserving neuron density in the penumbra region. Peruru et al. demonstrated that DCW could reduce carbonyl content and lipid peroxidation while improving the concentration of antioxidant enzymes such as superoxide dismutase, catalase, and glutathione, ultimately enhancing the survival rate of rat brain neurons (Peruru et al., 2020 ▶). Therefore, the increase in neuronal density in the penumbra after MCAO is achieved through the inactivation of inflammatory mediators by DCW. The interaction between the infarcted and penumbra regions among the injured groups further demonstrated that DCW could enhance the activity of penumbra nerve cells by maintaining synaptic plasticity between neurons to replace the damaged area (Appel et al., 2009 ▶). As a result, DCW improved animal movement performance and reduced infarct area by promoting neuronal recovery in the penumbra region. As previously mentioned, iridoid glycosides are the active components of DCW, and evidence suggests that the neuroprotective effect of DCW treatment correlates with the serum concentration of harpagoside (Chen et al., 2018 ▶; Li et al., 2015 ▶). Therefore, the reduction of infarct volume and the preservation of micro-anatomical structure can be attributed to the neuroprotective properties of harpagoside.

In conclusion, our study demonstrates that administering DCW prior to a stroke event can reduce the volume of infarcts and improve behavioral performance in rats. The observed neuroprotective effects of DCW may be attributed to its anti-inflammatory, analgesic, and antioxidant properties, mainly due to iridoid glycosides, flavonoids, and phenolic compounds. Our findings suggest that administering DCW at a dose of 400 mg/kg is particularly effective in preserving neuron density in the penumbra and reducing infarct volume after stroke. These results provide valuable insights into the potential application of DCW as a natural therapeutic agent for stroke treatment. Further research is necessary to elucidate the underlying mechanisms of DCW in limiting the infarct area and promoting neuronal recovery. Our study highlights the importance of exploring natural compounds like DCW to develop novel stroke therapies. 

## Conflicts of interest

The authors have declared that there is no conflict of interest. 

## References

[B1] Abroumand Gholami A, Gheybi F, Molavi AM, Tahmasebi F, Papi A, Babaloo H (2023). Effect of polycaprolactone/carbon nanotube scaffold implantation along with liposomal ellagic acid in hippocampal synaptogenesis after spinal cord injury. NMJ.

[B2] Appel K, Rose T, Fiebich B, Röhnert P, Claus D, Gerth A, Wilken D (2009). Neuroprotective and antiinflammatory effects of extracts from in vitro cultivated harpagophytum procumbens (Devil’s Claw). ZPT.

[B3] Ashioti M, John S, Andrew SB, Michel M, Andrew M, Michel MW, Steve CR (2009). Neither in vivo MRI nor behavioural assessment indicate therapeutic efficacy for a novel 5HT1A agonist in rat models of ischaemic stroke. BMC Neurol.

[B4] Azam AA, Ismail IS, Shaikh MF, Shaari K, Abas F (2019). Effects of Clinacanthus nutans leaf extract on lipopolysaccharide-induced neuroinflammation in rats: a behavioral and 1H NMR-based metabolomics study. Avicenna J Phytomed.

[B5] Chen C, Zhang H, Xu H, Xue R, Zheng Y, Wu T (2018). Harpagoside rescues the memory impairments in chronic cerebral hypoperfusion rats by inhibiting PTEN activity. J Alzheimer's Dis.

[B6] Ciancarelli I, Morone G, Iosa M, Cerasa A, Calabrò RS, Iolascon G (2022). Influence of oxidative stress and inflammation on nutritional status and neural plasticity: New perspectives on post-stroke neurorehabilitative outcome. J Nutr.

[B7] Fiebich BL, Muñoz E, Rose T, Weiss G, McGregor GP (2012). Molecular targets of the antiinflammatory Harpagophytum procumbens (devil’s claw): inhibition of TNFα and COX-2 gene expression by preventing activation of AP-1. Phytother Res.

[B8] García-Bonilla L, Rosell A, Torregrosa G, Salom JB, Alborch E, Gutiérrez M (2011). Guía de recomendaciones en la aplicación de modelos animales para el estudio del ictus. Neurologia.

[B9] Garcia JH, Wagner S, Liu K-F, Hu X (1995). Neurological deficit and extent of neuronal necrosis attributable to middle cerebral artery occlusion in rats: statistical validation. Stroke.

[B10] Georgiev MI, Alipieva KI, Denev P (2010). Antioxidant activity and bioactive constituents of the aerial parts of Harpagophytum procumbens plants. Biotechnol Biotechnol Equip.

[B11] Ghazavi H, Shirzad S, Forouzanfar F, Negah SS, Rad MR, Vafaee F (2020). The role of resveratrol as a natural modulator in glia activation in experimental models of stroke. Avicenna J Phytomed.

[B12] Grąbkowska R, Matkowski A, Grzegorczyk-Karolak I, Wysokińska H (2016). Callus cultures of Harpagophytum procumbens (Burch DC ex Meisn ; production of secondary metabolites and antioxidant activity. South African J Bot.

[B13] Huang TH-W, Tran VH, Duke RK, Tan S, Chrubasik S, Roufogalis BD (2006). Harpagoside suppresses lipopolysaccharide-induced iNOS and COX-2 expression through inhibition of NF-κB activation. J Pharm.

[B14] Jin T, Leng B (2022). Cynaropicrin averts the oxidative stress and neuroinflammation in ischemic/reperfusion injury through the modulation of NF-kB. Appl Biochem Biotechnol.

[B15] Kolpakova ME, Zubarevа AA, Artamonova TD, Lisovskaya EK, Chefu SG, Yagmurov OD (2017). Experimental model of osteonecrosis of the jaw in rats treated with zoledronic acid. Br J Oral Maxillofac Surg.

[B16] Koycheva IK, Mihaylova LV, Todorova MN, Balcheva-Sivenova ZP, Alipieva K, Ferrante C (2021). Leucosceptoside a from Devil’s Claw modulates psoriasis-like inflammation via suppression of the PI3K/AKT signaling pathway in keratinocytes. Molecules.

[B17] Li J, Ding X, Zhang R, Jiang W, Sun X, Xia Z (2015). Harpagoside ameliorates the amyloid-β-induced cognitive impairment in rats via up-regulating BDNF expression and MAPK/PI3K pathways. Neurosci J.

[B18] Lu J-D, Sun M-L, Wang X-P (2023). Butylphthalide protects against ischemia-reperfusion injury in rats via reducing neuron ferroptosis and oxidative stress. J Investig Med.

[B19] Mahomed IM, Ojewole JAO (2006). Anticonvulsant activity of Harpagophytum procumbens DC [Pedaliaceae] secondary root aqueous extract in mice. Brain Res Bull.

[B20] Mariano A, Bigioni I, Mattioli R, Di Sotto A, Leopizzi M, Garzoli S (2022). Harpagophytum procumbens root extract mediates anti-inflammatory effects in osteoarthritis synoviocytes through CB2 Activation. Pharmaceuticals (Basel).

[B21] McCrary MR, Jesson K, Wei ZZ, Logun M, Lenear C, Tan S (2020). Cortical transplantation of brain‐mimetic glycosaminoglycan scaffolds and neural progenitor cells promotes vascular regeneration and functional recovery after ischemic stroke in mice. Adv Healthc Mater.

[B22] Menghini L, Recinella L, Leone S, Chiavaroli A, Cicala C, Brunetti L (2019). Devil’s claw (Harpagophytum procumbens) and chronic inflammatory diseases: A concise overview on preclinical and clinical data. Phytother Res.

[B23] Peruru R, Usha Rani R, Thatiparthi J, Sampathi S, Dodoala S, Prasad KVSRG (2020). Devil’s claw (Harpagophytum procumbens) ameliorates the neurobehavioral changes and neurotoxicity in female rats exposed to arsenic. Heliyon.

[B24] Pulatov SS (2022). Efficacy of ipidacrine in the recovery period of ischaemic stroke. WBPH.

[B25] Sabahi M, Ahmadi SA, Kazemi A, Mehrpooya M, Khazaei M, Ranjbar A (2022). The effect of Oleoylethanolamide (OEA) add-on treatment on inflammatory, oxidative stress, lipid, and biochemical parameters in the acute ischemic stroke patients: randomized double-blind placebo-controlled study. Oxid Med Cell Longev.

[B26] Shirzad S, Tayaranian Marvian M, Abroumand Gholami A, Gharehbaghi M, Marefati N, Salmani H (2024). Unveiling the effects of left hemispheric intracerebral hemorrhage on long-term potentiation and inflammation in the Bilateral Hippocampus: A preclinical study. J Stroke Cerebrovasc Dis.

[B27] Stadler J, Schurr H, Doyle D, Garmo L, Srinageshwar B, Spencer MR (2022). Temporal profile of reactive astrocytes after ischemic stroke in rats. Neuroglia.

[B28] Sun X, Xiong Z, Zhang Y, Meng Y, Xu G, Xia Z (2012). Harpagoside attenuates MPTP/MPP+ induced dopaminergic neurodegeneration and movement disorder via elevating glial cell line‐derived neurotrophic factor. J Neurochem.

[B29] Wang Y, Luo Y, Yao Y, Ji Y, Feng L, Du F (2020). Silencing the lncRNA Maclpil in pro-inflammatory macrophages attenuates acute experimental ischemic stroke via LCP1 in mice. J Cereb Blood Flow Metab.

